# Supplementary material for: Non-Alcoholic Fatty Liver Disease Is Associated with a Decreased Catalase (CAT) Level, CT Genotypes and the T Allele of the -262 C/T CAT Polymorphism
Source: Cells. 2023 Sep 7;12(18):2228. doi: 10.3390/cells12182228 (PMC10527641; doi:10.3390/cells12182228)
Supplement: Supplementary file 1 [file cells-12-02228-s001.zip › cells-2494779-supplementary.pdf]

cells

MDPI

Article

Non-Alcoholic Fatty Liver Disease Is Associated with a Decreased Catalase (CAT) Level, CT Genotypes and the T Allele of the -262 C/T *CAT* Polymorphism.

Marcin Kosmalski [1,*], Izabela Szymczak-Pajor [2], Józef Drzewoski [3] and Agnieszka Śliwińska [2].

Table S1. Spearman correlations of CAT level and age, anthropometric, biochemical, BP, HSI and FLI parameters in +NAFLD and −NAFLD groups.

	**Group 0 −NAFLD (n=142)**		**Group 1 +NAFLD (n=139)**	
**Parameters***	**Rho******	**P****	**Rho******	**P****
Age [years]	0.0454	0.6392	-0.363	**0.00008**
BMI [kg/m2]	0.0783	0.4367	-0.062	0.5508
WC [cm]	0.2891	**0.0041**	-0.034	0.7435
HC [cm]	0.1152	0.261	-0.181	0.082
WHR	0.2862	**0.0045**	0.1252	0.2319
SBP [mmHg]	-0.021	0.832	-0.164	0.0833
DBP [mmHg]	-0.106	0.2727	-0.078	0.4125
Creatinine [µmol/l]	0.1762	0.0681	-0.088	0.3575
eGFR [ml/min/1,73m^2]	-0.016	0.8719	0.2819	**0.0027**
FPG [mmol/l]	0.1874	0.0555	0.0587	0.5465
PPG[mmol/l]	0.0838	0.4002	0.0324	0.737
HbA1c [%]	0.1032	0.3785	0.1982	0.0674
Uric acid [µmol/l]	-0.014	0.9105	-0.081	0.4861
TCH [mmol/l]	-0.088	0.3768	0.2946	**0.0022**

Cells **2023**, *12*, 2228. https://doi.org/10.3390/cells12182228 www.mdpi.com/journal/cells

LDL-CH [mmol/l]	-0.003	0.9745	0.2269	**0.0199**
HDL-CH [mmol/l]	-0.124	0.2135	0.1357	0.1697
TG [mmol/]	-0.026	0.7977	0.0216	0.8263
ALT [U/l]	0.253	**0.0099**	0.0874	0.3641
AST [U/l]	0.063	0.5269	-0.059	0.5395
Total bilirubin [μmol/l]	0.1467	0.1413	0.0833	0.3938
GGTP [U/l]	-0.028	0.7926	-0.003	0.9799
HSI	0.1371	0.183	0.0046	0.9649
FLI	0.1589	0.1539	-0.078	0.4911

* ALT – alanine aminotransferase, AST – aspartic aminotransferase, BMI – body mass index, BP- blood pressure, CAT- catalase, DBP – diastolic blood pressure, eGFR – estimated glomerular filtration rate, FLI – Fatty Liver Index, FPG – fasting plasma glucose, GGTP- gamma-glutamyltransferase, HbA1c – glycated hemoglobin, HDL-CH – HDL cholesterol, HC – hip circumference, HSI – Hepatic Steatosis Index, LDL-CH - LDL cholesterol, NAFLD – non-alcoholic fatty liver disease, PPG – postprandial plasma glucose, SBP – systolic blood pressure, TCH – total cholesterol, TG – triglycerides, WC − waist circumference, WHR – waist-hip ratio.
** p-value.
*** Rho - Spearman's rank correlation coefficient.
The bolded results indicate statistically significant differences.

Table S2. Anthropometric parameters, biochemical characteristics, BP, HSI, FLI in carriers of CC, CT, TT genotypes of -262 C/T *CAT* polymorphism in +NAFLD and −NAFLD groups.

**Parameter***	**Group 0 –NAFLD (n=142)**			**p****	**Group 1 +NAFLD (n=139)**			**p****	**p** -NAFLD vs. +NAFLD**
	**CC**	**CT**	**TT**		**CC**	**CT**	**TT**		
Age [years]	68.00(56.75; 76.25)	73.00 (62.00; 78.00)	58.00(53.00; 76.5)	0.1281	65.00(58.00; 74.00)	63.00(52.00; 74.00)	70.00(59.00;76.5)	0.4428	0.1023
WC [cm]	98.00(89.00; 106.00)	96.00(89.50; 108.5)	105(88.5; 122.5)	0.2722	108.00(100.3; 118.00)	106.00(102.00; 118.00)	106.00(100.00;113.50)	0.5935	**<0.001** D<br>**<0.01** E

HC [cm]	106.00(98.00; 110.00)	105.00(97.25; 111.80)	109.00(96.50; 114.00)	0.9460	110.00(105.00; 20.003)	110.00(103.00; 116.00)	117.00(108.50;120.00)	0.5089	**<0.001** D
BMI [kg/m^2]	27.47(23.80; 29.49)	27.64(23.59; 31.00)	29.70(23.93; 34.33)	0.4682	31.29(27.96; 34.33)	31.10(27.92; 35.49)	30.49(28.40;34.69)	0.9761	**<0.001** D **<0.05** E
WHR	0.9333(0.8812; 0.9811)	0.9412(0.8656; 0.9812)	0.9633(0.9073; 1.0910)	0.3980	0.9732(0.9238;1.0360)	0.9667(0.9123; 1.0290)	0.9327(0.8841;0.9498)	0.0722	**<0.05** D
SBP [mmHg]	130(120; 140)	130(120; 150)	120(120; 135)	0.2699	130(120; 150)	140(130; 150)	160(130;190)	0.0568	0.1230
DBP [mmHg]	80(70; 80)	80(70; 90)	70(65; 90)	0.4034	80(70; 90)	80(70; 90)	90(80;100)	0.0943	0.1830
FPG [mmol/l]	6.870(5.255; 11.2)	6.235(4.518; 8.078)	5.015(4.655; 8.435)	0.0573	7.9(5.433; 11.4)	8.060(5.610; 9.872)	8.500(6.185; 12.9)	0.8480	0.0532
PPG[mmol/l]	8.280(5.905; 15.52)	7.120(5.443; 13.35)	10 (8.375; 15.33)	0.1227	9.45(6.063; 14.67)	9.885(6.553; 16.14)	9.8(8.07; 12.68)	0.7807	0.4291
HbA1c [%]	7.67(6.02; 9.185)	7.425(6.155; 9.083)	6.320(5.788; 9.065)	0.8057	8.575(6.695; 9.995)	8.935(7.0; 10.24)	7.88(6.205; 10.96)	0.8653	0.3006
ALT [U/l]	19(13; 28.25)	19(14.5; 30)	24(11; 60.5)	0.9281	25(18.5; 42.5)	25(16; 30)	27(13.5; 31.5)	0.6516	**<0.01 D**
AST [U/l]	20(15; 27.25)	21(16.5; 35.5)	19(15; 63)	0.4601	23(17; 35)	23(17; 35)	18(15.5; 25)	0.3677	0.2357
GGTP [U/l]	22(16.36; 48.49)	27.9(16.18; 52.3)	19(14.5; 25)	0.4853	41(24; 76)	45.87(24.92; 75)	49(40.28; 66)	0.9731	**<0.001 D**
Total bilirubin [μmol/l]	8.525(6.948; 12.88)	8.65(6.698; 12.91)	7.95(7.115; 11)	0.8147	10.2(7.56; 15.10)	11.23(8.375; 17.10)	9.8(7.075; 17.39)	0.6729	0.1265
Creatinine [μmol/l]	76.00(61.50; 99.00)	74.00(62.00;101.00)	67.00(63.00;92.00)	0.0853	73.5(64.00;90)	78.00(68.00;95.00)	73.00(56.50;95.50)	0.6304	0.9186
eGFR [ml/min/1,73m^2]	84(55; 103.5)	92(53; 100)	108(70; 114.5)	0.4677	82(62; 103.3)	77(65; 100)	72(65; 100.5)	0.9537	0.8691
TCH [mmol/l]	4.2(3.24; 5.05)	4.425(3.595; 5.768)	4.1(3.46; 5.275)	0.5225	4.4(3.6; 5.4)	4.6(3.835; 5.350)	4.53(4.185; 5.57)	0.8532	0.569
LDL-CH [mmol/l]	2.5(1.715; 3.3)	2.68(2.125; 3.625)	2.6(1.76; 3.82)	0.4399	2.55(1.8; 3.4)	2.78(2.12; 3.5)	2.82(2.63; 3.42)	0.3922	0.5967
HDL-CH [mmol/l]	1.09(0.89; 1.395)	1.12(0.945; 1.35)	1.12(1.035; 1.315)	0.7652	0.99(0.76; 1.38)	1.05(0.93; 1.32)	1.01(0.865; 1.8)	0.5649	0.7503
TG [mmol/]	1.22(0.825; 1.58)	1.21(0.835; 1.79)	1.14(0.97; 1.57)	0.8431	1.84(1.153; 3.035)	1.865(1.23; 2.34)	1.62(1.075; 2.845)	0.8362	**<0.001D** **<0.05 E**
Uric acid [μmol/l]	276.5(235; 318)	276(249; 387)	263(206.5; 404)	0.5465	355(268.5; 451.5)	349(287.5; 403.5)	378(307; 437)	0.8794	**<0.01D**

HSI	30.85(29.28; 32.7)	31.5(29.3; 32.95)	32.2(29.75; 34.25)	0.4076	43.15(39; 48.83)	42.95(37.85; 50.23)	44.2(40.5; 48.75)	0.7870	**<0.001 D** **<0.001E** **<0.05F**
FLI	33.65(20.99; 52.58)	29.76(19.83; 52.87)	34.04(24.47; 59.36)	0.6766	83.45(75.23; 96.05)	91.82(75.83; 96.8)	81.44(78.13; 90.33)	0.6931	**<0.001D** **<0.001E** **<0.05F**

* ALT – alanine aminotransferase, AST – aspartic aminotransferase, BMI – body mass index, BP – blood pressure, DBP – diastolic blood pressure, eGFR – estimated glomerular filtration rate, FLI – Fatty Liver Index, FPG – fasting plasma glucose, GGTP- gamma-glutamyltransferase, HbA1c – glycated hemoglobin, HC – hip circumference, HDL-CH – HDL cholesterol, HSI – Hepatic Steatosis Index, LDL-CH - LDL cholesterol, NAFLD – non-alcoholic fatty liver disease, PPG – postprandial plasma glucose, SBP – systolic blood pressure, TCH – total cholesterol, TG – triglycerides, WC- waist circumference, WHR – waist-hip ratio.

**p-value assessed using the Manna-Whitney U test and Kruskal-Wallis test with Dunn`s multiple comparison test.

Data is expressed as median (Quartile 1; Quartile 3).

The bolded results indicate statistically significant differences.

A- CC vs CT
B- CC vs TT
C- CT vs TT
D- −NAFLD CC vs +NAFLD CC
E- −NAFLD CT vs +NAFLD CT
F- −NAFLD TT vs +NAFLD TT
G- −NAFLD CT+TT vs +NAFLD CT+TT

Table S3. Anthropometric parameters, biochemical characteristics, BP, HSI, FLI in in carriers of C or T alleles of -262 C/T *CAT* polymorphism in +NAFLD and −NAFLD groups.

**Parameter***	**Group 0 −NAFLD (n=142)**		**p****	**Group 1 +NAFLD (n=139)**		**p****	**P** −NAFLD vs. +NAFLD**
	**CC**	**CT+TT**		**CC**	**CT+TT**		
Age [years]	68(56.75; 76.25)	71.5(60; 78)	0.4175A	65(58; 74)	64(53.25; 74)	0.5234A	0.4389B 0.0537C

WC [cm]	98(89; 106)	98(90; 112)	0.6470A	108(100.3; 118)	106(102; 116.8)	0.5062A	**<0.001B**<br>**<0.01C**
HC [cm]	106(98; 110)	105(98; 112)	0.9566A	110(105; 120)	111.5(103.3; 119.3)	0.8214A	**<0.001B**<br>**<0.01C**
BMI [kg/m^2]	27.47(23.8; 29.49)	27.68(23.64; 31.48)	0.3416A	31.29(27.96; 34.33)	31.06(28.08; 34.84)	0.8311A	**<0.001B**<br>**<0.001C**
WHR	0.9333(0.8812; 0.9811)	0.9412(0.8776; 0.9891)	0.8072A	0.9732(0.9238; 1.036)	0.9606(0.9051; 1.018)	0.4249A	**<0.01B**<br>0.2587C
SBP [mmHg]	130(120; 140)	130(120; 147.5)	0.4522A	130(120; 150)	140(130; 150)	0.0912A	0.4583B<br>0.0739C
DBP [mmHg]	80(70; 80)	80(70; 88.75)	0.8173A	80(70; 90)	80(70; 90)	0.2811A	0.7114B<br>0.3180C
FPG [mmol/l]	6.87(5.255; 11.2)	6.07(4.625; 8.078)	**<0.05**A	7.9(5.433; 11.4)	8.12(5.85; 10)	0.9691A	0.3693B<br>**<0.01C**
PPG[mmol/l]	8.28(5.905; 15.52)	7.73(6.02; 13.66)	0.5011A	9.45(6.063; 14.67)	9.8(6.63; 15.63)	0.5290A	0.8960B<br>0.2484C
HbA1c [%]	7.67(6.02; 9.185)	7.25(6.013; 9.055)	0.7259A	8.575(6.695; 9.995)	8.26(6.82; 10.22)	0.8215A	0.0945B<br>0.1121C
ALT [U/l]	19(13; 28.25)	19.5(12.75; 32)	0.7647A	25(18.5; 42.5)	25.5(16; 30.75)	0.3672A	**<0.001B**<br>0.2036C
AST [U/l]	20(15; 27.25)	21(16.75; 35.25)	0.2381A	23(17; 35)	22.5(16.25; 31)	0.3624A	**<0.05B**<br>0.9124C
GGTP [U/l]	22(16.36; 48.49)	23.47(16.39; 41.75)	0.9821A	41(24; 76)	46.34(29; 72)	0.9176A	**<0.001B**<br>**<0.001C**
Total bilirubin [µmol/l]	8.525(6.948; 12.88)	8.5(6.79; 11.75)	0.8259A	10.2(7.56; 15.1)	11.2(8.3; 17)	0.3988A	0.1194B<br>**<0.05C**
Creatinine [µmol/l]	76(61.5; 99)	72.5(63; 101)	0.9602A	73.5(64; 90)	78(67.25; 94.75)	0.5129A	0.9934 B

							0.5302C
eGFR [ml/min/1,73m^2]	84(55; 103.5)	84(56; 108)	0.9691A	82(62; 103.3)	77(65.5; 100)	0.9037A	0.8556B<br>0.9256C
TCH [mmol/l]	4.2(3.24; 5.05)	4.35(3.6; 5.7)	0.3475A	4.4(3.6; 5.4)	4.59(3.945; 5.415)	0.8515A	0.1453 B<br>0.6409C
LDL-CH [mmol/l]	2.5(1.715; 3.3)	2.675(2.1; 3.588)	0.2196A	2.55(1.8; 3.4)	2.8(2.19; 3.48)	0.2284A	0.9930B<br>0.6931C
HDL-CH [mmol/l]	1.09(0.89; 1.395)	1.12(1.013; 1.308)	0.5803A	0.99(0.76; 1.38)	1.045(0.93; 1.32)	0.3110A	0.3406B<br>0.3975C
TG [mmol/]	1.22(0.825; 1.58)	1.175(0.9; 1.7)	0.5642A	1.84(1.153; 3.035)	1.79(1.23; 2.34)	0.6077A	**<0.001B**<br>**<0.001C**
Uric acid [µmol/l]	276.5(235; 318)	275(247; 390)	0.3872A	355(268.5; 451.5)	349(292; 413)	0.6274A	**<0.001B**<br>**<0.05C**
HSI	30.85(29.28; 32.7)	31.55(29.45; 33.28)	0.4107A	43.15(39; 48.83)	43.1(38.8; 50.2)	0.9605A	**<0.001B**<br>**<0.001C**
FLI	33.65(20.99; 52.58)	30.32(21.29; 58.35)	0.9327A	83.45(75.23; 96.05)	87.99(76.92; 95.56)	0.7442A	**<0.001B**<br>**<0.001C**

* ALT – alanine aminotransferase, AST – aspartic aminotransferase, BMI – body mass index, BP – blood pressure, DBP – diastolic blood pressure, eGFR – estimated glomerular filtration rate, FLI – Fatty Liver Index, FPG – fasting plasma glucose, GGTP- gamma-glutamyltransferase, HbA1c – glycated hemoglobin, HC – hip circumference, HDL-CH – HDL cholesterol, HSI – Hepatic Steatosis Index, LDL-CH − LDL cholesterol, NAFLD – non-alcoholic fatty liver disease, PPG – postprandial plasma glucose, SBP – systolic blood pressure, TCH – total cholesterol, TG – triglycerides, WC- waist circumference, WHR – waist-hip ratio.

**p-value assessed using the Manna-Whitney U test and Kruskal-Wallis test with Dunn`s multiple comparison test.

Data is expressed as median (Quartile 1; Quartile 3).

The bolded results indicate statistically significant differences.

A - CC vs. CT+TT

B - −NAFLD CC vs. +NAFLD CC

C - −NAFLD CT+TT vs. +NAFLD CT+TT

Table S4. Spearman correlations between CAT level and age, anthropometric and biochemical parameters in patients without NAFLD (−NAFLD group; n=142) and in patients with NAFLD (+NAFLD group; n=139) adjusted to CC, CT, TT -262 C/T *CAT* polymorphism distribution.

	Group 0 −NAFLD (n=142)						Group 1 +NAFLD (n=139)					
	CC		CT		TT		CC		CT		TT	
**Parameters***	**Rho*** **	**P****	**Rho*** **	**P****	**Rho*** **	**P****	**Rho*** **	**P****	**Rho*** **	**P****	**Rho*** **	**P****
Age [years]	-0,019	0,8708	-0,002	0,9934	-0,071	0,9063	-0,268	**0,0335**	-0,555	**0,0001**	0,1622	0,7325
BMI [kg/m2]	-0,011	0,9302	-0,034	0,879	0,2143	0,6615	0,017	0,9018	-0,263	0,1386	0,6071	0,1667
WC [cm]	0,2253	0,0667	0,2472	0,2556	0,4286	0,3536	-0,031	0,8277	-0,103	0,5619	0,5766	0,1873
HC [cm]	0,055	0,6587	0,1689	0,4412	0,0714	0,9063	0,0168	0,9061	-0,571	**0,0004**	0,5045	0,2548
SBP [mmHg]	-0,08	0,5009	0,0712	0,7189	-0,019	0,8952	-0,098	0,4456	-0,289	0,0639	0,0364	0,9508
DBP [mmHg]	-0,209	0,0754	0,0988	0,617	-0,094	0,7381	-0,053	0,6799	-0,084	0,5989	-0,073	0,8508
WHR	0,2452	0,0455	0,347	0,1047	0,6071	0,1667	-0,051	0,7204	0,3485	**0,0434**	0,75	0,0663
Creatinine [μmol/l]	0,1365	0,2494	0,1778	0,3654	0,4505	0,3159	-0,099	0,4431	-0,208	0,1855	0,5714	0,2
eGFR [ml/min/1,73m²]	0,0308	0,7959	-0,102	0,6071	-0,071	0,9063	0,2367	0,064	0,4383	**0,0037**	-0,595	0,1532
FPG [mmol/l]	0,1421	0,2339	0,4128	**0,0324**	0,3143	0,5639	-0,073	0,5798	0,3676	**0,018**	-0,179	0,7131
PPG[mmol/l]	0,1208	0,3227	0,0922	0,6474	-0,071	0,9063	-0,046	0,7207	0,2065	0,1953	-0,786	0,048
HbA1c [%]	0,1678	0,2441	0,0474	0,8473	-0,086	0,9194	0,1819	0,2264	0,3127	0,0764	-0,464	0,3024
Uric acid [μmol/l]	-0,099	0,527	0,1177	0,6621	0,0286	1	-0,003	0,9866	-0,204	0,2717	0,7	0,2333

TCH [mmol/l]	-0,107	0,3809	0,1075	0,5936	-0,214	0,6615	0,2659	**0,0418**	0,4215	**0,0068**	-0,5	0,2667
LDL-CH [mmol/l]	0,0135	0,9121	0,0092	0,9643	-0,25	0,5948	0,2326	0,0762	0,2977	0,0657	-0,321	0,4976
HDL-CH [mmol/l]	-0,097	0,4275	-0,085	0,6804	-0,631	0,123	0,282	**0,0305**	-0,128	0,4438	-0,036	0,919
TG [mmol/]	-0,021	0,8643	0,0349	0,8657	-0,357	0,4444	-0,105	0,4243	0,3167	**0,0495**	-0,429	0,3536
ALT [U/l]	0,3715	**0,0015**	0,0472	0,8188	0,0714	0,9063	0,0514	0,694	0,2175	0,1665	0,1261	0,7976
AST [U/l]	0,1783	0,1397	-0,295	0,1429	-0,143	0,7825	-0,112	0,3921	0,0868	0,5847	-0,054	0,8889
Total bilirubin [μmol/l]	0,1169	0,3426	0,0739	0,7142	0,0714	0,9063	0,1086	0,4131	0,0418	0,7952	-0,607	0,1667
GGTP [U/l]	0,058	0,6571	-0,269	0,2042	-0,321	0,4976	-0,232	0,077	0,5034	**0,0018**	0,3571	0,4444
HSI	0,0936	0,4513	0,0627	0,7817	0,3571	0,4444	0,074	0,6023	-0,111	0,5379	0,5714	0,2
FLI	0,1703	0,2054	-0,094	0,7109	0,25	0,5948	-0,161	0,2794	0,0612	0,7665	0,6071	0,1667

* ALT – alanine aminotransferase, AST – aspartic aminotransferase, BMI – body mass index, DBP – diastolic blood pressure, eGFR – estimated glomerular filtration rate, FLI – Fatty Liver Index, FPG – fasting plasma glucose, GGTP- gamma-glutamyltransferase, HbA1c – glycated hemoglobin, HDL-CH – HDL cholesterol, HC – hip circumference, HSI – Hepatic Steatosis Index, LDL-CH − LDL cholesterol, NAFLD – non-alcoholic fatty liver disease, PPG – postprandial plasma glucose, SBP – systolic blood pressure, T-CH – total cholesterol, TG – triglycerides, WC − waist circumference, WHR – waist-hip ratio.
** p-value.
*** Rho - Spearman's rank correlation coefficient.
The bolded results indicate statistically significant differences.

Table S5. Spearman correlations between CAT concentration and anthropometric and biochemical parameters in patients without NAFLD (−NAFLD group; n=142) and in patients with NAFLD (+NAFLD group; n=139) adjusted to allele distribution of -262 C/T polymorphism of *CAT*.

	**Group 0 −NAFLD (n=142)**				**Group 1 +NAFLD (n=139)**			
	**CC**		**CT+TT**		**CC**		**CT+TT**	
Parameters*	**Rho*****	**P****	**Rho*****	**P****	**Rho*****	**P****	**Rho*****	**P****
Age [years]	-0,019	0,8708	0,0865	0,6211	-0,268	**0,0335**	-0,544	**0,0001**

BMI [kg/m2]	-0,011	0,9302	0,0687	0,7181	0,017	0,9018	-0,175	0,2795
WC [cm]	0,2253	0,0667	0,2905	0,1194	-0,031	0,8277	-0,012	0,9392
HC [cm]	0,055	0,6587	0,1573	0,4064	0,0168	0,9061	-0,481	**0,0015**
WHR	0,2452	**0,0455**	0,3865	**0,0349**	-0,051	0,7204	0,3763	**0,0153**
Creatinine [µmol/l]	0,1365	0,2494	0,2649	0,1241	-0,099	0,4431	-0,128	0,3814
eGFR [ml/min/1,73m^2]	0,0308	0,7959	-0,113	0,5174	0,2367	0,064	0,3781	**0,0074**
FPG [mmol/l]	0,1421	0,2339	0,3418	0,0516	-0,073	0,5798	0,229	0,1174
PPG[mmol/l]	0,1208	0,3227	-0,019	0,9167	-0,046	0,7207	0,1117	0,4499
HbA1c [%]	0,1678	0,2441	-0,022	0,9157	0,1819	0,2264	0,1981	0,2203
Uric acid [µmol/l]	-0,099	0,527	0,0921	0,6837	-0,003	0,9866	-0,202	0,2381
TCH [mmol/l]	-0,107	0,3809	-0,017	0,9255	0,2659	**0,0418**	0,3238	**0,0264**
LDL-CH [mmol/l]	0,0135	0,9121	-0,06	0,7414	0,2326	0,0762	0,2154	0,1506
HDL-CH [mmol/l]	-0,097	0,4275	-0,185	0,3021	0,282	**0,0305**	-0,119	0,4356
TG [mmol/]	-0,021	0,8643	-0,027	0,8833	-0,105	0,4243	0,2292	0,1255
SBP [mmHg]	-0,08	0,5009	0,0987	0,5728	-0,098	0,4456	-0,297	**0,0382**
DBP [mmHg]	-0,209	0,0754	0,1166	0,5047	-0,053	0,6799	-0,138	0,3443
ALT [U/l]	0,3715	**0,0015**	0,0146	0,9359	0,0514	0,694	0,1572	0,2808
AST [U/l]	0,1783	0,1397	-0,21	0,2413	-0,112	0,3921	0,1381	0,344
Total bilirubin [µmol/l]	0,1169	0,3426	0,1672	0,3447	0,1086	0,4131	0,0328	0,8249
GGTP [U/l]	0,058	0,6571	-0,254	0,1688	-0,232	0,077	0,4905	**0,0008**
HSI	0,0936	0,4513	0,1022	0,5977	0,074	0,6023	-0,115	0,4785
FLI	0,1703	0,2054	0,0346	0,8695	-0,161	0,2794	0,1427	0,4282

* ALT – alanine aminotransferase, AST – aspartic aminotransferase, BMI – body mass index, DBP – diastolic blood pressure, eGFR – estimated glomerular filtration rate, FLI – Fatty Liver Index, FPG – fasting plasma glucose, GGTP- gamma-glutamyltransferase, HbA1c – glycated hemoglobin, HDL-CH – HDL cholesterol, HC – hip circumference, HSI – Hepatic Steatosis Index, LDL-CH - LDL cholesterol, NAFLD – non-alcoholic fatty liver disease, PPG – postprandial plasma glucose, SBP – systolic blood pressure, TCH – total cholesterol, TG – triglycerides, WC – waist circumference, WHR – waist-hip ratio.

** p-value.

*** Rho – Spearman's rank correlation coefficient.

The bolded results indicate statistically significant differences.

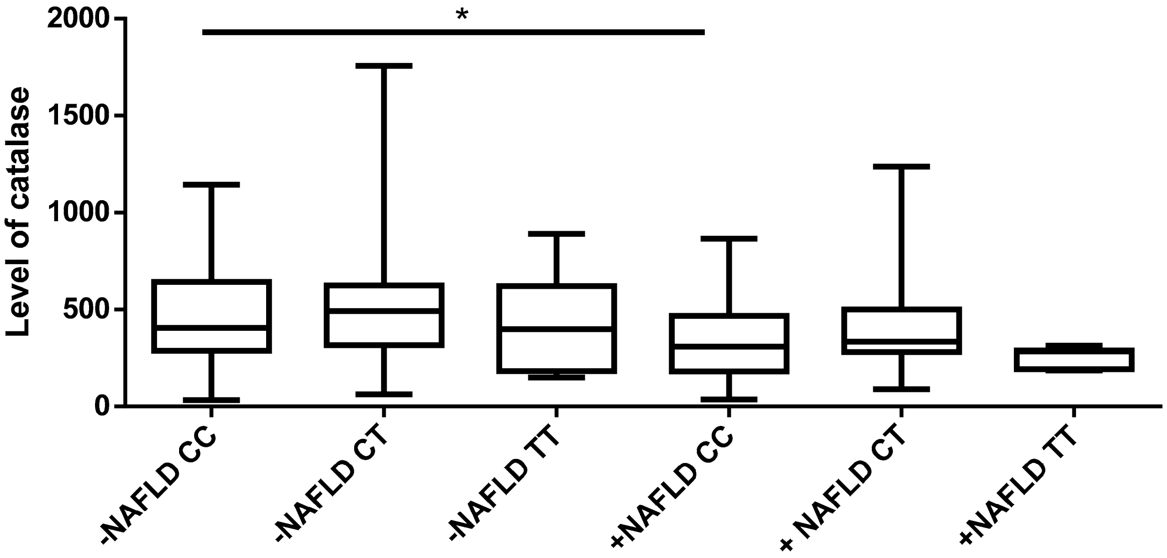

Figure S1. The level of CAT in patients with NAFLD (+NAFLD) and without NAFLD (−NAFLD) divided accordingly to CC, CT, TT genotype carrier of the -262 C/T *CAT* polymorphism, measured by ELISA. The data are expressed as medians with lower and upper quartiles and minimal and maximal values. *p<0.05 – NAFLD group vs. +NAFLD group.

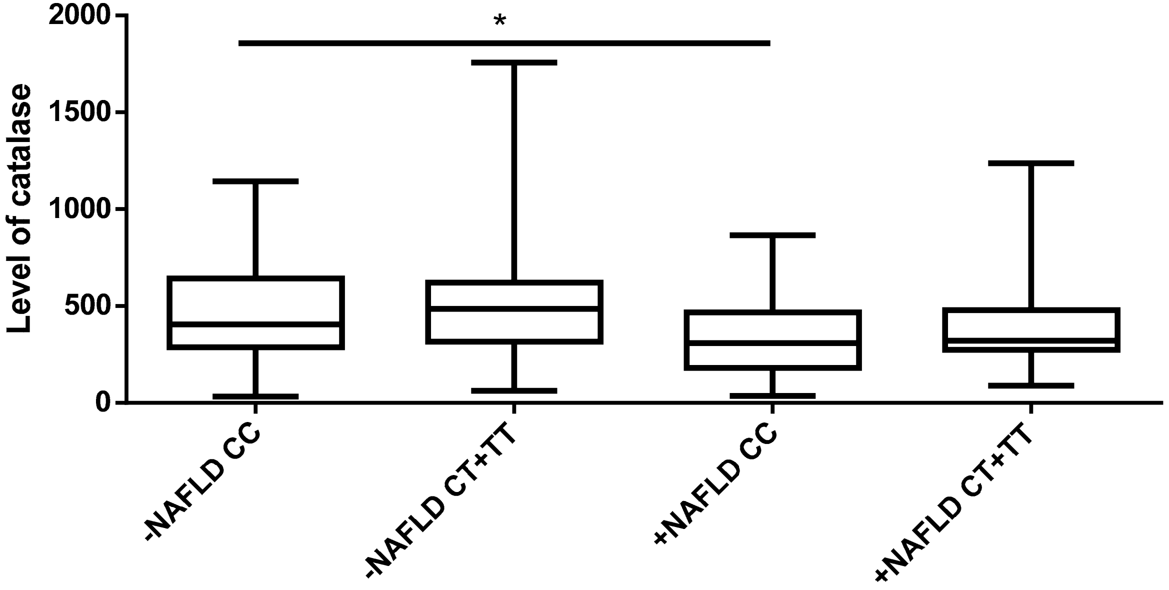

Figure S2. The level of CAT in patients with NAFLD (+NAFLD) and without NAFLD (−NAFLD) divided accordingly to C, T alleles carrier of the -262 C/T *CAT* polymorphism, measured by ELISA. The data are expressed as medians with lower and upper quartiles and minimal and maximal values. *p<0.05 −NAFLD group vs. +NAFLD group.
